# Anterior segment indices in mentally retarded children

**DOI:** 10.1038/s41598-023-41827-6

**Published:** 2023-09-04

**Authors:** Hassan Hashemi, Amin Mohayeji, Abdollah Farzaneh, Abbasali Yekta, Hadi Ostadimoghaddam, Amir Asharlous, Mehdi Khabazkhoob

**Affiliations:** 1 Noor Research Center for Ophthalmic Epidemiology, Noor Eye Hospital, Tehran, Iran; 2 Noor Ophthalmology Research Center, Noor Eye Hospital, Tehran, Iran; 3 Rehabilitation Research Center, Department of Optometry, School of Rehabilitation Sciences, Iran University of Medical Sciences, Tehran, Iran; 4 Department of Optometry, Mashhad University of Medical Sciences, Mashhad, Iran; 5 Refractive Errors Research Center, Mashhad University of Medical Sciences, Mashhad, Iran; 6 Department of Basic Sciences, School of Nursing and Midwifery, Shahid Beheshti University of Medical Sciences, Tehran, Iran

**Keywords:** Anatomy, Diseases, Medical research, Signs and symptoms

## Abstract

To compare the anterior segment indices between mentally retarded and normal children. The current study was conducted as a cohort. In this study, 73 mentally retarded and 76 normal children were selected from normal school and special schools for mentally retarded children using random cluster sampling method. Mental retardation in children was confirmed by a psychologist. Optometry examinations including visual acuity and refraction were performed for all participants, and ultimately, corneal imaging measurements were taken by Pentacam. The mean age of mentally retarded and normal children was of 13.30 ± 1.83 and 13.05 ± 1.82 years, respectively (P = 0.180). A multiple generalized estimating equations model demonstrated that there is a significant association between central corneal thickness (CCT) (coef = 1.011, P < 0.001), corneal diameter (CD) (coef = 0.444, P = 0.046), anterior chamber depth (ACD) (coef = 0.23), P < 0.001) and index of vertical asymmetry (IVA) (coef = 0.12, P < 0.001) and mental retardation. Cerebral palsy children had higher keratoconus index (KI), central keratoconus index (CKI), index of height asymmetry(IHA), and index of height decentration (IHD) compared to those without cerebral palsy (P < 0.05). Children with moderate mental retardation had higher index of surface variance (ISV), IVA, IHA, and IHD than those with mild mental retardation (P < 0.05). The mean and standard deviation of CCT, CD, ACD and IVA index in mentally retarded children were 535.3 ± 46.68 micron, 11.87 ± 0.42 mm, 3.29 ± 0.24 mm and 0.25 ± 0.18 mm, respectively. These indices in the normal group were 525.53 ± 47.52 micron, 11.84 ± 0.38 mm, 3.15 ± 0.28 mm and 0.17 ± 0.05 mm, respectively. The findings of this study showed that some anterior segment indices were different in mentally retarded compared to normal children. Moreover, some keratoconus indicators were worse in cerebral palsy children and children with higher grade mental retardation. So, it is important to consider keratoconus screening in these children.

## Introduction

Mental retardation is defined as an incomplete mental development that leads to a fundamental limitation in general mental ability, intellectual performance, adaptive behavior, and functional skills compared to people of the same age, same sex, and same socio-cultural background. These limitations are observed in various aspects such as communication with others, personal care, independence and academic skills. The global prevalence of this disorder was reported 10.37 people per 1000 people in 2011^1,2^. This disorder can be either inherited or acquired, former being merely responsible for more than 1700 types of mental retardation-related disorders in the world^1^.

In general, beside other associated problems, ocular problems have also been reported to affect these children so that studies show that more than half of mentally retarded children manifest ocular problems. It has also been reported that ocular problems increase with growing intensity of mental retardation. Refractive errors are among the most common ocular problems in these individuals^3–6^.

Corneal formation in embryonic period is influenced by different genes. Therefore, genetic problems in humans can have different consequences on corneal morphology^7^. Since genetics plays a significant role in causing mental retardation and also genetics has an effect on the morphology and shape of the cornea, there is a possibility that genetic problems cause differences in corneal tomographic and topographic indices between mentally retarded and normal individual. Also, due to the prevalence of ocular problems in these individuals, corneal topography and tomography indices should be evaluated.

Mentally retarded children usually do not cooperate properly for subjective tests. Therefore, most decisions are based on objective findings. Nowadays, modern imaging devices are used to evaluate the corneal health and refractive power which assess the corneal topography and tomography indices. These indices give us more detailed information about the surface of the cornea as well as its layers, which are used for pre-surgical evaluations^8^.

Although previous studies showed that there are some differences between normal population and individuals with Down’s syndrome in terms of corneal indices, there are limited studies regarding the evaluation of these indices in mentally retarded children. We do not have access to corneal status of these subjects which is necessary in their contact lens fitting as well as refractive surgery procedures. Hence, it is of great importance to have comprehensive information about the topographic and tomographic condition of the cornea of these individuals. Therefore, the aim of the current study is to compare the anterior segment indices between mentally retarded and normal children.

## Methods

Considering that aim of the current study was to evaluate the effect of mental retardation on corneal indices, it was conducted as a cohort. This cohort study was carried out in Tehran in 2020. The sample population was from the students of Tehran schools and special schools for mentally retarded children of Tehran. In this study, sampling was performed by random cluster sampling from both types of schools.

Mentally retarded children were randomly selected from two schools in Tehran.

All children with an IQ lower than 70 were considered mentally retarded. Cases with an IQ between 55 and 69 and between 40 and 54 were considered as mild and moderate mental retardation, respectively. Children with severe mental retardation (IQ < 40) were not examined due to poor cooperation.

Normal children were also randomly selected from the closest schools to these two aforementioned schools. Inclusion criteria were as follows: psychologist's confirmation of the mental retardation, absence of Down syndrome and no history of previous ocular surgeries. Poor cooperation for imaging procedure, accompanying systemic disease and positive history of ocular trauma and ocular surgery were the exclusion criteria. Following sample selection and coordination with the parents, informed consent was obtained from the parents or guardians before the procedures, and all the examinations were performed in an appointed day in the school.

For all the participants, optometric examinations including visual acuity measurement with LED visual acuity chart (Smart LC 13, Medizs Inc., Korea) at a distance of 6 m and non-cycloplegic auto-refraction was performed by an auto-refractometer/keratometer (ARK-510A, Nidek Co. LTD, Aichi, Japan). The subjective refraction was performed to determine the optimal distance optical correction and the best-corrected distance visual acuity (BCVA) was recorded.

Corneal imaging using Pentacam Oculus (Pentacam HR, Oculus, Germany) was performed by one person under the same conditions. To avoid the effect of diurnal variation, all imaging was performed between 10 a.m. and 15 p.m. and at least 3 h after waking up. The Pentacam indices examined in this study were: keratometry, central corneal thickness, anterior chamber depth, anterior chamber volume, anterior chamber angle, corneal volume, corneal diameter and keratoconus indices.

In this study, descriptive and analytical data were reported after entering the data into SPSS software. Considering the correlation of the two eyes, generalized estimating equations (GEE) analysis was used to compare the variables between the two groups. We also used multiple GEE model to control the correlation effect of fellow eyes as well as potential confounders including age and sex.

### Ethical consideration

The study was conducted in accordance with the Helsinki Declaration. All procedures involving children were approved by the Ethics Committee of Iran University of Medical Sciences. Written informed consent was obtained from the students’ parents/legal guardians and oral consent was obtained from all students (ethical code: IR.IUMS.REC.1401.186).

## Results

In the current study, 76 normal and 73 mentally retarded individuals were selected.

Of the 298 eyes examined, one eye was excluded from the analysis due to the Pentacam measurement error, and the final analysis was performed on 297 eyes. 43 of mentally retarded children had cerebral palsy (CP). Moreover, 55.9% and 44.1% of mentally retarded children had mild and moderate mental retardation, respectively.

48.7% of mentally retarded subjects and 59.3% of normal group were males (P = 0.081). All the participants were in the age range of 9–18 years with an average age of 13.30 ± 1.83 and 13.05 ± 1.82 years in the mentally retarded and in the normal groups respectively (P = 0.180).

The mean spherical equivalent was − 0.88 ± 1.97 D in mentally retarded children and − 0.54 ± 1.61 D in the normal group; there was no statistically significant difference in SE between the two groups (P = 0.108).

As shown in Table 1, except for central corneal thickness, corneal diameter, and anterior chamber angle, statistically significant differences were observed in other variables between the two groups (P < 0.05). The mean keratometry, anterior chamber depth, anterior chamber volume, and corneal volume are higher in the mentally retarded children group than in the normal group. As can be seen in this table, all keratoconus indices between the two groups have statistically significant differences and these indices are higher in the mentally retarded group than in the normal group.

**Table 1:** The mean and standard deviation of anterior segment indices in mentally retarded and normal children.

Cornea indices	Normal	Mental retardation	P-value*
	Mean ± SD	Mean ± SD	
Mean keratometry (diopter)	43.46 ± 1.56	44.05 ± 2.03	0.014
Minimum keratometry (diopter)	42.91 ± 1.56	43.26 ± 1.91	0.123
Maximum keratometry (diopter)	44.00 ± 1.64	44.84 ± 2.46	0.003
Corneal astigmatism (diopter)	1.09 ± 0.66	1.57 ± 1.67	0.004
Central corneal thickness (micron)	525.53 ± 47.52	535.3 ± 46.68	0.098
Corneal diameter (mm)	11.84 ± 0.38	11.87 ± 0.42	0.553
Anterior chamber depth (mm)	3.15 ± 0.28	3.29 ± 0.24	< 0.001
Anterior chamber volume (mm^3^)	190.32 ± 33.19	198.28 ± 29.52	0.039
Anterior chamber angle (degree)	39.9 ± 4.95	40.82 ± 5.35	0.131
Corneal volume	59.3 ± 4.73	60.64 ± 4.64	0.023
Index of surface variance	21.13 ± 4.18	31.24 ± 19.65	< 0.001
Index of vertical asymmetry	0.17 ± 0.05	0.25 ± 0.18	< 0.001
Keratoconus index	1.03 ± 0.02	1.05 ± 0.06	< 0.001
Central keratoconus index	1.01 ± 0.01	1.01 ± 0.02	< 0.001
Index of height asymmetry	5.26 ± 4.07	9.23 ± 11.51	< 0.001
Index of height decentration	0.01 ± 0.01	0.02 ± 0.02	< 0.001

The P-value was calculated by generalized estimating equations model.

**Figure 1:**
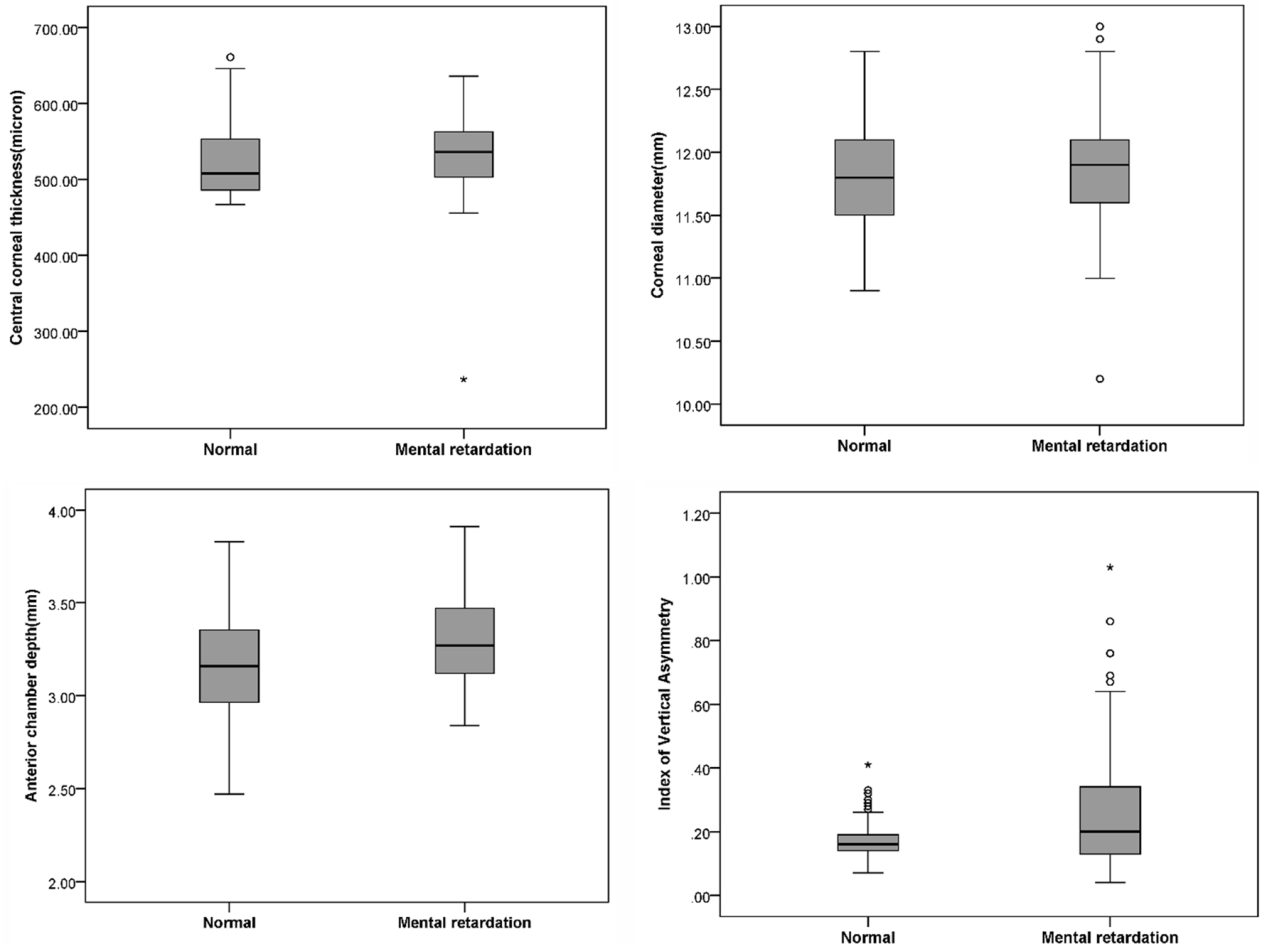
The distribution of keratoconus indices in mentally retarded and normal children.

The association between topography indices and keratoconus indices with mental retardation was evaluated by a GEE multiple model. The multiple model was run using the backward method and included all studied variables along with age and sex. The results of this model showed that among the mentioned variables, corneal central thickness (coef = 1.011, P < 0.001), corneal diameter (coef = 0.444, P < 0.046), anterior chamber depth (coef = 0.23), P < 0.001) and IVA index (coef = 0.12, P < 0.001) had a direct and significant association with mental retardation. Figure 1 presents the distribution of theses indices in two groups.

Table 2 compares examined indices between mentally retarded children with and without CP. As seen in Table 2, children with CP had higher keratoconus index, central keratoconus index, index of height asymmetry, and index of height decentration compared to children without CP. Comparison between mild and moderate mentally retarded cases (Table 3) showed that the index of surface variance, index of vertical asymmetry, index of height asymmetry, and index of height decentration were significantly higher in children with moderate mental retardation than those with mild mental retardation.

**Table 2:** The mean and standard deviation of anterior segment indices in mentally retarded children with and without cerebral palsy.

	Without cerebral palsy	With cerebral palsy	P-value*
	Mean ± SD	Mean ± SD	
Mean keratometry (diopter)	43.73 ± 1.86	44.26 ± 2.13	0.178
Minimum keratometry (diopter)	42.95 ± 1.97	43.48 ± 1.84	0.165
Maximum keratometry (diopter)	44.52 ± 2.13	45.05 ± 2.65	0.255
Corneal astigmatism (diopter)	1.57 ± 1.72	1.57 ± 1.65	0.997
Central corneal thickness (micron)	536.81 ± 54.67	534.26 ± 40.62	0.773
Corneal diameter (mm)	11.82 ± 0.43	11.90 ± 0.41	0.266
Anterior chamber depth (mm)	3.28 ± 0.23	3.30 ± 0.25	0.773
Anterior chamber volume (mm^3^)	197.42 ± 30.20	198.86 ± 29.21	0.780
Anterior chamber angle (degree)	40.18 ± 5.06	41.26 ± 5.52	0.203
Corneal volume	60.14 ± 5.23	60.98 ± 4.19	0.347
Index of surface variance	29.8 ± 18.50	32.23 ± 20.44	0.470
Index of vertical asymmetry	0.23 ± 0.16	0.27 ± 0.19	0.200
Keratoconus index	1.03 ± 0.04	1.06 ± 0.07	0.013
Central keratoconus index	1.01 ± 0.01	1.02 ± 0.02	0.046
Index of height asymmetry	6.35 ± 4.78	11.2 ± 14.11	0.015
Index of height decentration	0.02 ± 0.02	0.03 ± 0.03	0.029

The P-value was calculated by generalized estimating equations model.

**Table 3:** The mean and standard deviation of anterior segment indices in mild and moderate mentally retarded children.

	Mild mentally retardation	Moderate mentally retardation	P-value*
	Mean ± SD	Mean ± SD	
Mean keratometry (diopter)	44.00 ± 1.87	44.12 ± 2.24	0.768
Minimum keratometry (diopter)	43.36 ± 1.90	43.14 ± 1.92	0.561
Maximum keratometry (diopter)	44.63 ± 2.03	45.09 ± 2.91	0.365
Corneal astigmatism (diopter)	1.27 ± 1.22	1.95 ± 2.06	0.045
Central corneal thickness (micron)	531.74 ± 51.41	539.8 ± 39.86	0.320
Corneal diameter (mm)	11.84 ± 0.43	11.9 ± 0.41	0.420
Anterior chamber depth (mm)	3.29 ± 0.23	3.29 ± 0.26	0.936
Anterior chamber volume (mm^3^)	200.23 ± 28.06	195.8 ± 31.32	0.411
Anterior chamber angle (degree)	41.23 ± 5.43	40.3 ± 5.24	0.288
Corneal volume	60.55 ± 5.22	60.75 ± 3.83	0.800
Index of surface variance	26.60 ± 15.16	37.11 ± 22.97	0.002
Index of vertical asymmetry	0.21 ± 0.15	0.31 ± 0.20	0.002
Keratoconus index	1.05 ± 0.05	1.05 ± 0.07	0.501
Central keratoconus index	1.01 ± 0.01	1.02 ± 0.02	0.101
Index of height asymmetry	6.85 ± 6.66	12.23 ± 15.16	0.032
Index of height decentration	0.02 ± 0.01	0.03 ± 0.03	0.006

## Discussion

The importance of this study is the evaluation of corneal topography and tomography indices in mentally retarded children, except for Down syndrome, which has not been conducted so far.

According to the multiple GEE model in the present study, there was no statistically significant difference in most of the corneal topographic and tomographic parameters between the two groups of normal and mentally retarded children. However, the difference between four parameters of central corneal thickness, corneal diameter, anterior chamber depth and IVA index between two groups of normal and mentally retarded subjects was statistically significant.

It should be noted that the results of the simple GEE model were different from the multiple model. Since the multiple model considers all variables and controls the effect of confounders, it has more valid results. Therefore, our discussion is based on multiple model results.

Regarding these observed changes, considering that the amount of changes compared to the standard deviation index of the mentioned parameters are smaller numbers and also because these changes are not significant from a clinical point of view, it can be said that the topographic and tomographic features of the cornea in mentally challenged children are not significantly different from normal people. However, we discuss some points regarding the differences found.

The findings indicated that the cornea was thicker in the mentally retarded group than in the normal group of children. According to studies, three similar studies, including the studies of Akinci et al.^9^, Cumurcu et al.^10^, and Karadag et al.^11^ have reported a thicker cornea in mentally retarded children. The results of the current study are consistent with the findings of these studies in terms of corneal thickness in mentally retarded individuals^9–11^.

Studies have also been conducted involving patients with Down syndrome; including the studies of Hashemi et al.^12^, Alio et al.^13^, Karakucuk et al.^14^, Aslan et al.^15^, and Evereklioglu et al.^16^, which reported a difference in corneal thickness (Table 4).

**Table 4:** Comparison of central corneal thickness between children with some challenges and normal.

Author	Challenge	With challenge	Normal	P-value
Akinci^9^	Mental retardation	549.7 ± 21.4	521.6 ± 16.9	< 0.001
Cumurcu^10^	Cerebral palsy	568.46 ± 31.94	549.53 ± 26.16	< 0.001
Karadag^11^	Mental retardation	554 ± 39.7	535.7 ± 24.2	< 0.05
Hashemi^12^	Down syndrome	516.7 ± 33	555.7 ± 33.1	< 0.001
Alio^13^	Down syndrome	503 ± 28.11	545 ± 32.56	< 0.001
Karakucuk^14^	Down syndrome	524.1 ± 33.4	546.1 ± 27.7	< 0.001
Aslan^15^	Down syndrome	494.27 ± 47	539.3 ± 40	< 0.001
Evereklioglu^16^	Down syndrome	488.39 ± 39.87	536.25 ± 20.70	< 0.001

It is worth mentioning that the differences related to the amount of corneal thickness in similar studies might be due to the differences in the type of disorder, population, ethnical and racial discrepancies, as well as the measurement methods. For instance, in the study of Karadag et al.^11^, corneal thickness has been evaluated using an ultra-sonographic device, whereas, in our study, Pentacam device was used for this purpose.

According to the studies conducted to date, no reason has been found justifying the increase in the corneal thickness of mentally retarded people. In the study of Cumurcu et al.^10^, no statistically significant difference was found in the corneal thickness between the two groups of cerebral palsy with mental retardation and cerebral palsy without mental retardation. However, in this study, it was mentioned that the increase in corneal thickness in the group of combined cerebral palsy and mental retardation might indicate that mental retardation itself can be a factor in increasing the central corneal thickness^10^.

The thicker cornea of mentally retarded children in our study, who mostly had cerebral palsy, is probably due to the fact that the normal growth and development of the body is disturbed in cerebral palsy. Subsequently, the changes in the corneal thickness also do not follow their normal developmental course^17^. The human corneal thickness decreases significantly in the first year of life^18^. Therefore, disruption in the normal growth of the cornea can cause the cornea to remain thicker in people with cerebral palsy. The observed differences in corneal thickness, corneal diameter, anterior chamber depth and IVA index between mentally retarted and normal children clinically imply that some consideration should be taken in screening glaucoma or keratoconus in the mentally retarted children. Intraocular pressure, corneal biomechanical indices, and OCT analysis of the optic nerve head in mentally retarted children are important relevant issue that could be the topics of future studies.

In addition to the aforementioned changes, the variable values of anterior chamber depth are also higher in mentally retarded children than the control group. No study has been conducted to investigate this index in children with cerebral palsy so far. It seems that genetic and environmental factors have an effect on this parameter. Further studies are warranted to investigate this particular issue.

As it was mentioned, the IVA index in mentally retarded children was significantly higher than normal children. Hashemi et al. stated that the best index for detecting keratoconus is the IVA index, in which the values higher than 0.2 microns is considered as an indicator of keratokonus^19^. In our study, the average IVA index in mentally retarded people is equal to 0.25 microns, which indicates the presence of subclinical keratoconus. Since the rest of the indicators for diagnosing keratoconus are not present in these individuals, it seems that the increase in the IVA index in these people may be due to structural changes in the cornea and does not indicate ectatic changes in the cornea. In future studies, it is recommended to use corneal biomechanical index along with corneal irregularity indices to detect keratoconus.

Nevertheless, screening for keratoconus is recommended in mentally retarded children considering they have higher IVA.

The corneal diameter (i.e. limbus to limbus diameter) of mentally retarded children, is statistically significantly higher than normal children. However, from a clinical point of view, the difference is not significant (0.1 mm).Indeed, the lack of axial length data does not allow us to make a correct judgment in this regard, and more studies are needed for this matter since this index (corneal diameter) is important in some clinical measures such as prescribing contact lenses.

In general, to justify these significant differences in the mentioned indices, there might be two possible explanations. The first reason is that the mentally retarded children in our study mostly had cerebral palsy. Since the natural growth and development of the body is disturbed in cerebral palsy, it can induce an effect on the natural growth and development of the eye and its components^17^. This means that the structure of the cornea and anterior chamber, etc., are likely to undergo changes. Examining the anatomical dimensions of the eye in these people seems an interesting topic. Considering this hypothesis, the difference in corneal thickness, anterior chamber depth, IVA index and corneal diameter can be justified to some extent.

The second reason that can be cited is the existence of a genetic background in the development of cerebral palsy. Probably, this genetic factor can also affect ocular structure. According to studies, approximately 14% of cerebral palsy etiologies are genetic problems^20^. Genetic background can also play an important role in creating changes in the cornea and ocular structure.

In the current study, an attempt has been made to examine all the important corneal topography and tomography indices in mentally retarded children. However, this study also has limitations. Children with cerebral palsy were also considered as mentally retarded and were included in this category. Not taking into account intraocular pressure (IOP) and corneal biomechanical indices, the lack of diversity of disorders in the mentally retarded group are among other the limitations of this study.

According to the results of the present study, CP children had higher keratoconus index, central keratoconus index, index of height asymmetry, and index of height decentration compared to those without CP. In addition, cases with moderate mental retardation had higher index of surface variance, index of vertical asymmetry, index of height asymmetry, and index of height decentration than those with mild mental retardation. Other ocular problems such as vision disorders, strabismus and retinal problems have already received attention in these children^6,21,22^. Our finding indicates the necessity of more attention to CP children and children with more severe mental retardation in terms of keratoconus indicators^23^. It is recommended that these children have regular visits for keratoconus workup.

Finally, it is suggested that in future studies, the three groups of cerebral palsy, Down syndrome and normal people be compared with each other, and in addition to the indices used in the current study, intraocular pressure and corneal biomechanics also be considered.

The findings of this study showed that some anterior segment indices were different in mentally retarded compared to normal children. Moreover, some keratoconus indicators were worse in CP children and children with higher grade mental retardation. So, it is important to consider keratoconus screening in these children.

## Data Availability

The datasets used and/or analyzed during the current study available from the corresponding author on reasonable request.

## References

[1] Maia N, NabaisSá MJ, Melo-Pires M, de Brouwer APM, Jorge P (2021). Intellectual disability genomics: Current state, pitfalls and future challenges. BMC Genom..

[2] Maulik PK, Mascarenhas MN, Mathers CD, Dua T, Saxena S (2011). Prevalence of intellectual disability: A meta-analysis of population-based studies. Res. Dev. Disabil..

[3] Joshi RS, Somani AA (2013). Ocular disorder in children with mental retardation. Indian J. Psychiatry.

[4] Warburg M (2001). Visual impairment in adult people with intellectual disability: Literature review. J. Intellect. Disabil. Res. JIDR.

[5] Muhit M, Karim T, Jahan I, Al Imam MH, Das MC, Khandaker G (2022). Epidemiology of eye diseases among children with disability in rural Bangladesh: A population-based cohort study. Dev. Med. Child Neurol..

[6] Heydarian S, Abbasabadi MM, Khabazkhoob M, Hoseini-Yazdi H, Gharib M (2022). Vision abnormalities in children and young adults with cerebral palsy; a systematic review. Semin. Ophthalmol..

[7] Miesfeld JB, Brown NL (2019). Eye organogenesis: A hierarchical view of ocular development. Curr. Top. Dev. Biol..

[8] Feng MT, Belin MW, Ambrósio R, Grewal SP, Yan W, Shaheen MS, Jordon CA, McGhee C, Maeda N, Neuhann TH (2011). International values of corneal elevation in normal subjects by rotating Scheimpflug camera. J. Cataract Refract. Surg..

[9] Akinci A, Oner O, Munir K (2010). Central corneal thickness in children with intellectual disability: A controlled study. Cornea.

[10] Cumurcu T, Cumurcu BE, Kilic R, Ozturk B, Etikan I (2009). Increased central corneal thickness in children with cerebral palsy. Cornea.

[11] Karadag R, Erdurmus M, Yagci R, Keskin UC, Hepsen IF, Durmus M (2007). Central corneal thickness in individuals with intellectual disabilities. Cornea.

[12] Hashemi H, Makateb A, Mehravaran S, Fotouhi A, Shariati F, Asgari S (2020). Mapping the corneal thickness and volume in patients with Down syndrome: A comparative population-based study. Arq. Bras. Oftalmol..

[13] Alio JL, Vega-Estrada A, Sanz P, Osman AA, Kamal AM, Mamoon A, Soliman H (2018). Corneal morphologic characteristics in patients with Down syndrome. JAMA Ophthalmol..

[14] Karakucuk Y, Altinkaynak H, Comez A, Beyoglu A (2020). Objective evaluation of corneal and lens densitometry in children with Down syndrome. J. Fr. Ophtalmol..

[15] Aslan L, Aslankurt M, Yüksel E, Özdemir M, Aksakal E, Gümüşalan Y, Özdemir G (2013). Corneal thickness measured by Scheimpflug imaging in children with Down syndrome. J. AAPOS..

[16] Evereklioglu C, Yilmaz K, Bekir NA (2002). Decreased central corneal thickness in children with Down syndrome. J. Pediatr. Ophthalmol. Strabismus.

[17] Andrew MJ, Sullivan PB (2010). Growth in cerebral palsy. Nutr. Clin. Pract..

[18] Maripudi S, Byrd J, Qureshi A, Stoleru G, Levin MR, Saeedi OJ, Munir W, Bazemore M, Karwoski B, Martinez C (2020). Pediatric corneal structural development during childhood characterized by ultrasound biomicroscopy. J. Pediatr. Ophthalmol. Strabismus.

[19] Hashemi H, Beiranvand A, Yekta A, Maleki A, Yazdani N, Khabazkhoob M (2016). Pentacam top indices for diagnosing subclinical and definite keratoconus. J. Curr. Ophthalmol..

[20] Jin SC, Lewis SA, Bakhtiari S, Zeng X, Sierant MC, Shetty S, Nordlie SM, Elie A, Corbett MA, Norton BY (2020). Mutations disrupting neuritogenesis genes confer risk for cerebral palsy. Nat. Genet..

[21] Rauchenzauner M, Schiller K, Honold M, Baldissera I, Biedermann R, Tschiderer B, Albrecht U, Arnold C, Rostasy K (2021). Visual impairment and functional classification in children with cerebral palsy. Neuropediatrics.

[22] Jacobson L, Lennartsson F, Nilsson M (2020). Retinal ganglion cell topography predicts visual field function in spastic cerebral palsy. Dev. Med. Child Neurol..

[23] Hashemi H, Asgari S (2022). Corneal characteristics in Down syndrome patients with normal and keratoconic cornea. Front. Med..

